# Long-term low-dose aspirin promotes laser-induced choroidal neovascularization through suppressing TSP-1 expression

**DOI:** 10.3389/fncel.2025.1716229

**Published:** 2025-12-05

**Authors:** Caijiao Yi, Chang Luo, Jiawu Zhao, Christophe Roubeix, Judith Lechner, Rosana Penalva, Nan Yang, Jian Liu, Qichang Wang, Usha Chakravarthy, Florian Sennlaub, Mei Chen, Heping Xu

**Affiliations:** 1 AIER Eye Institute, Changsha Aier Eye Hospital, Changsha, Hunan, China; 2 The Wellcome-Wolfson Institute for Experimental Medicine, Queen’s University Belfast, Belfast, United Kingdom; 3 Sorbonne University, INSERM, CNRS, Institut de la Vision, Paris, France

**Keywords:** age-related macular degeneration, angiogenesis, inflammation, aspirin, macrophages

## Abstract

**Purpose:**

To investigate the impact of low-dose, long-term aspirin use on neovascular age-related macular degeneration (nAMD).

**Methods:**

Adult C57BL/6J or Thbs-1^–/–^ mice were treated with daily aspirin (1.25 mg/kg) for 8 weeks before being subjected to laser-induced choroidal neovascularization (CNV). The animals were left for 7–10 days with continued aspirin use before the eyes were collected for further investigations. Bone marrow-derived macrophages (BMDMs) and primary retinal pigment epithelial (RPE) cells were treated with different concentrations of aspirin (1, 10, 100 μM) for two days before being subjected to LPS+IFNγ for 16 h. The expression of cytokine genes was evaluated by qRT-PCR. The concentrations of thrombospondin-1 (TSP-1) were measured by ELISA.

**Results:**

Aspirin treatment did not affect circulating immune cell profiles in normal mice but significantly increased CD11b^+^ cells in laser-induced CNV mice. The treatment significantly increased the severity of laser-induced CNV and reduced serum levels of TSP-1. *In vitro* aspirin treatment upregulated *Tnfa* and *Ccl22*, down-regulated *Thbs-1* mRNA expression, and reduced TSP-1 production in LPS+IFNγ-treated M1 BMDMs but not RPE cells. Thbs-1^–/–^ mice developed severe laser-induced CNV, which was not affected by aspirin intervention. nAMD patients had significantly lower serum levels of TSP-1 than healthy controls, although no significant difference was found between nAMD patients with and without aspirin use.

**Conclusion:**

Low-dose long-term aspirin use promoted the severity of laser-induced CNV by down-regulating TSP-1. Lower serum levels of TSP-1 may be a risk factor for nAMD. The long-term ocular safety of aspirin should be validated in prospective cohorts.

## Introduction

Age-related macular degeneration (AMD) is the major cause of blindness in the elderly in developed countries, affecting nearly 200 million people globally (Wong et al., 2014). Early AMD is characterized by the deposition of lipid-rich waste materials (Drusen) underneath the retinal pigment epithelial (RPE) cells of the macula, and the disease can progress into intermediate (larger Drusen > 125 μm) and advanced stages (Fleckenstein et al., 2021). There are two advanced forms of AMD: dry (geographic atrophy, GA) and wet (neovascular AMD, nAMD). GA is due to the progressive death of macular cells, including RPE and light-detecting neurons (photoreceptors). Two complement inhibitors have been approved for GA to reduce disease progression (Dascalu et al., 2024; Chew, 2023). Wet AMD (nAMD) is the growth of abnormal blood vessels that are friable and leaky with consequential hemorrhage and/or leakage of fluid components, which, in turn, results in distortion and destruction of the outer retinal layers and causes severe visual dysfunction. Intravitreal injection of vascular endothelial cell growth factor (VEGF) inhibitors is the mainstream therapy for nAMD (Fleckenstein et al., 2021). Although anti-VEGF therapy can improve visual function in the short to medium term in nAMD through restoration of competence to the leaking vessels, visual function continues to decline due to the onset of fibrosis and atrophy, thus making it important to prevent nAMD through a better understanding of its pathogenesis.

AMD is a multi-factor disease. Genetic predisposition, aging, and environmental factors all play a role in the development and progression of AMD. Lifestyle, including physical activity, food, diet, and nutrition, can affect the onset or progression of AMD. For example, the AREDS and AREDS2 studies demonstrated that vitamins C and E, β-carotene and zinc supplementation reduced the progression in some patients to advanced AMD by 25% in 5 years (Broadhead et al., 2023; Chew et al., 2022). In addition to food supplements, the elderly often take various medications such as lipid-lowering drugs, beta-blockers or ACE inhibitors for age-related health conditions. Aspirin, as an anticoagulant, is frequently prescribed to the elderly at a low dose as a daily medication to reduce the risk of cardiovascular diseases. Early studies have reported a small but statistically significant association between Aspirin use and early AMD (Li et al., 2015; Kahawita and Casson, 2015). Others, including the AREDS or ARDES2 study (Keenan et al., 2019) found no association between aspirin use and AMD progression (Zhu et al., 2013; Cheung et al., 2013), but aspirin use was found to increase the risk of nAMD (Ye et al., 2014; Liew et al., 2013; de Jong et al., 2012). More recent studies have found that the association was affected by the duration of aspirin use. Regular aspirin use for 10 years (Klein et al., 2012) and over (Yan et al., 2022) but not for 5 years (Rim et al., 2019) was associated with a significant increase in the incidence of late and nAMD. The inconsistent results from population-based studies may be attributed to multiple factors such as the general health condition of the study population, the dose and duration of aspirin, and other medications. To address the issue, we explored the effect of low-dose (1.25 mg/kg daily, equivalent to 75 mg/adult/daily), and long-term (8 weeks, equivalent to 6–8 years of human age) aspirin use in the mouse model of laser-induced choroidal neovascularization (CNV) and investigated the mechanism of action.

## Materials and methods

### Animals and aspirin treatment

Between 8 and 12 weeks old C57BL/6J or Thbs-1^–/–^ mice were obtained from the Jackson Laboratories. The animals were maintained at the Biological Service Unit at Queen’s University Belfast or the animal facility of Aier Eye Institute under specific pathogen-free conditions in a 12/12-h light/dark cycle with free access to water and food. Aspirin (20 mg) was dissolved in ethanol (500 μL) as a stock solution and further diluted with water and adjusted to pH = 7.35∼7.45 immediately before use. Mice were treated with aspirin or vehicle (100 μL of 0.1% ethanol) via daily gavage feeding (30 μg/100 μL/mouse, ∼1.25 mg/kg body weight) or in drinking water (18 μg/mL) for 8 weeks before and 7–10 days after laser-induced CNV. For drinking water aspirin administration, fresh-prepared aspirin was changed every day. All animal studies were approved by the Animal Welfare Ethical Review Body of Queen’s University Belfast or Aier Eye Institute. The procedures complied with the UK Home Office Animals (Scientific Procedure) Act 1986, and the Association for Research in Vision and Ophthalmology (ARVO) statement for the Use of Animals in Ophthalmic and Vision Research.

### Laser-induced choroidal neovascularization

Laser coagulations were performed using a protocol described previously, with slight modifications (Lambert et al., 2013; Chen et al., 2016). In brief, mice were anesthetized with ketamine hydrochloride (60 mg/kg) (Fort George Animal Centre, Southampton, United Kingdom) and xylazine (5 mg/kg) (Pharmacia and Veterinary Products, Kiel, Germany) via intraperitoneal injection, and pupils were dilated with 0.5% tropicamide and 0.5% phenylephrine (Santen Pharmaceutical, Japan). Four laser burns per eye were created using laser photocoagulation (Haag Streit BM 900 slit Lamp and Argon Laser, Hagg Streit, Harlow, United Kingdom). Laser settings were 100 μm spot size, 250 mW power and 100 ms duration. Mice were euthanized through carbon dioxide (CO_2_) inhalation anesthesia followed by cervical dislocation 10 days after laser-induced CNV, gas flow was controlled within 30–70% of the chamber volume per minute and maintained for > 60 s after respiratory arrest.

### RPE/choroidal flatmount staining

Eyes were removed and fixed for 30 min at room temperature in 4% paraformaldehyde. After several washes in PBS, the eyes were dissected under a dissecting microscope, the cornea and lens were removed, and the retina was carefully detached from the RPE/choroid. The RPE/choroid tissues were incubated overnight with primary antibodies (biotinylated Griffonia Simplicifolia Lectin I-Isolectin B4 (1:100, Vector Laboratories Ltd., Peterborough, United Kingdom); rabbit anti-mouse collagen IV (1/100, ABD Serotec Ltd., Oxford, United Kingdom); CD102 (1/200, BD Pharmingen); mouse anti-TSP1 (1/200, Abcam); rabbit anti-IBA1 (1/200, Wako-Chem) in PBS supplemented with 0.5% Triton X-100, followed by incubation with appropriate Alexa-coupled secondary antibodies (Life Technologies) and Hoechst 1/1,000. The tissues were then flatmounted and examined by a fluorescent microscope (DM550B, Leica). The images were analyzed with ImageJ software.

### Flow cytometry

50 μL of whole blood was collected from D10 post-CNV mice and processed for immunostaining as described previously by us (Zhao et al., 2014). In brief, samples were incubated with Fcγ receptor blocker (rat anti-mouse CD16/CD32), followed by incubating with 50 μL of fluorochrome-conjugated antibody cocktail (Table 1) for 40 min on ice. The samples were washed with FACS buffer, re-suspended in 200 μL of FACS buffer, and processed for FACS analysis using the BD FACSCantoII (BD Biosciences, Oxford, United Kingdom). The data were analyzed by FlowJo Software (TreeStar, Inc., Ashland, OR, United States).

**TABLE 1:** Fluorochrome-conjugated antibodies used in flow cytometry.

Target molecule	Conjugated fluorochrome	Origin	Catalog no.	Dilution	Company
CD11b	PE-Cy7	Rat	552850	1:100	BD Bioscience
CD3	FITC	Rat	561798	1:100	BD Bioscience
Gr-1	PE	Rat	553128	1:100	BD Bioscience
Icam-1	FITC	Rat	557444	1:100	BD Bioscience
LFA-1	BV650	Rat	740453	1:100	BD Bioscience
CD62L	BV605	Rat	104438	1:100	BioLegend
PSGL-1	BV421	Rat	562807	1:100	BD Bioscience
MHC II	APC	Rat	17-5321-82	1:100	eBioscience

### Bone marrow-derived macrophage culture and treatment

Bone marrow-derived macrophages (BMDMs) were cultured from mouse tibias and femurs using the protocol described previously (Luo et al., 2012). Bone marrows were flushed out with Dulbecco’s modification of Eagle’s medium (DMEM, Gibco BRL, Grand Island, NY, United States) and red blood cells were removed with lysis buffer (0.75% NH4Cl, 0.02% Tris–HCl, pH 7.2) on ice for 15 min. Bone marrow cells were cultured in a DMEM supplement with 15% fetal calf serum (FCS, Gibco) and 15% L929 conditioned medium and seeded in 75-mm^2^ flasks at 37 °C in a 5% CO_2_ incubator for 5 days. The phenotype was confirmed by flow cytometry, and 96% of cells were CD11b^+^F4/80^+^.

BMDMs were seeded in six-well plates at a density of 1 × 10^6^ cells/well overnight. Cells were then activated as follows: M1 with IFN-γ (100 ng/mL, R&D Systems, Minneapolis, MN) and LPS (50 ng/mL, Sigma-Aldrich); M2 with IL-4 (20 ng/mL, R&D Systems) at 37 °C for 16 h. Before M1 or M2 polarization, BMDMs were pretreated with aspirin (10 μM) for 1 h (short-term) or 48 h (long-term).

### Mouse RPE cell culture and treatment

RPE cells were isolated and cultured from 8-week-old mouse eyes using a previously described method with slight modification (Chen et al., 2007). In brief, after the anterior segment of the eye and the lens were removed, eyecups were incubated with 0.5%(w/v) trypsin-EDTA (Gibco, Massachusetts, United States) at 37°C for 0.5 h. The cells were then removed by gentle aspiration. After washing, single-cell suspensions were cultured in DMEM containing 15% FCS in the first passage and 10% FCS for the rest passages. The phenotype was confirmed by RPE65 staining. Passages 3–5 were used in the study.

For aspirin treatment, mouse primary RPE cells were pretreated with different concentrations of aspirin (1, 10, 100 μM) for 48 h followed by 16 h of LPS (50 ng/mL) incubation. The cells were then processed for qRT-PCR and supernatants were collected for ELISA.

### Real-time RT-PCR

The total RNAs were extracted from BMDMs or RPE cells by RNeasy Mini Kit (Qiagen, Dusseldorf, Germany) according to the manufacturer’s instructions and cDNA was synthesized using the SuperScript II Reverse Transcriptase kit (Invitrogen, Paisley, United Kingdom). Murine mRNA expression levels were quantified by real-time PCR using the LightCycler 480 system with SYBR Green I Master (Roche Diagnostics GmbH, Mannheim, Germany). The primers used are listed in Table 2; 18S was used as a housekeeping gene. Gene fold changes were calculated by dividing the normalized values of treated samples by the normalized values of control samples.

**TABLE 2:** Primer sequences of mouse genes for RT-PCR.

Gene	Forward sequence 5′–3′	Reverse sequence 5′–3′
*iNOS*	GGCAAACCCAAGGT CTACGTT	TCGCTCAAGTTC AGCTTGGT
*Il-18*	GGCCCAGGAACAAT GGCT	TCATATCCTCGAACA CAGGC
*Tnfa*	TCTCATGCACCACCATCA AGGACT	ACCACTCTCCCTTTGCA GAACTCA
*IL-1*β	TCCTTGTGCAAGTG TCTGAAGC	ATGAGTGATACTGC CTGCCTGA
*Ccl2*	AGGTCCCTGTCATGC TTCTG	TCTGAACCCATTCC TTCTTG
*Ccl22*	AAGACAGTATCTGCTG CCAGG	GATCGGCACAGATA TCTCGG
*Cxcl2*	CCAGACAGAAGTCAT AGCCAC	GGCACATCAGGTAC GATCCA
*Cxcl1*	TGGCTGGGATTCACC TCAAG	TCTCCGTTACTTGGGG ACAC
*Thbs1*	TGTGGACTTCAGCGGT ACCTTCTT	GGACTGGGTGACTT GTTTCCACAT
*Vegfa*	GCACTGGACCCTGG CTTTA	CTTGATCACTTGATG GGACTTCTG
*Ym-1*	ACTTTGATGGCCTCA ACCTG	AATGATTCCTGCTCC TGTGG
*Arg-1*	TTATCGGAGCGCCTTT CTCAA	TGGTCTCTCACGTCATA CTCTGT
*Tgfb1*	GTGTGGAGCAACATGT GGAACT	GGGCTGATCCCGT TGATTTC
*Il-10*	TGCAGGACTTTAAGGGT TACTTGG	GGCCTTGTAGAC ACCTTGGTC
*18S*	GGCCCAGGAACAA TGGCT	TCATATCCTCGA ACACAGGC

### Measurement of thrombospondin-1 (TSP-1)

The study was approved by the Research Ethics Committee of Queen’s University Belfast and procedures were performed under the tenets of the Declaration of Helsinki on research involving human volunteers. Participants were recruited from the macular disease clinics in Belfast (Belfast Health and Social Care Trust, United Kingdom), with written informed consent obtained from each participant. Questionnaires were used to ascertain medical history (AMD, cardiovascular disease, hypertension and diabetes), medication history (cardiovascular medication, vitamins and low-dose aspirin) and clinical examination of participants. The questionnaire didn’t collect information on patients’ compliance or the duration of medication. Participants with systemic inflammatory or autoimmune diseases (e.g., active rheumatoid arthritis or active chronic bronchitis) and those undergoing steroid therapy or chemotherapy were excluded from the study. Participant samples were assigned randomly for experimental analysis. In the current study, 213 participant samples were analyzed, including 170 nAMD and 43 controls. Sixty-four of the nAMD patients were taking aspirin, and 106 were not.

The samples were collected in our previous study (Lechner et al., 2017). Venous blood (5 ml) was collected in tubes containing serum clot activator and centrifuged at 2,000 *g* for 15 min within 3 h of collection. After centrifugation, the serum was aliquoted and stored at −80 °C until analysis. TSP-1 or VEGF levels in the serum from nAMD patients and controls were measured using a human TSP-1 ELISA kit (Invitrogen, Carlsbad, CA, United States) or cytometric bead array (CBA) with CBA Flex Sets (BD Biosciences, Oxford, United Kingdom) following the manufacturer’s instructions.

### Mouse TSP-1 ELISA

The TSP-1 levels in the serum of mice and the supernatants from BMDMs and RPE cells with aspirin-treated were measured using a TSP-1 ELISA kit (P35441, CUSABIO, China) according to the manufacturer’s instructions. In brief, serum or supernatants were loaded into 96-well plates coated with TSP-1-capturing antibodies and incubated for 2 h at room temperature. After washing, 100 μL of anti-TSP-1 antibody conjugated with horseradish peroxidase (HRP) was added and incubated for 30 min at room temperature, followed by 100 μL of substrate incubation. The plate was read at 450 nm using a microplate reader (Tecan, Switzerland). The levels of TSP-1 were normalized by the total protein of the supernatants (determined by BCA assay, Cat: PC0020, Solarbio, Beijing, China).

### Statistical analysis

IBM SPSS Statistics (Version 25, IBM SPSS, Chicago, United States) and GraphPad Prism (Version 10, GraphPad Software, San Diego, CA) were used for statistical analysis. The normal distribution of continuous variables was assessed using the Shapiro–Wilk test, and the variance homogeneity was evaluated using the Levene test. The difference between two groups was compared using the Welch’s *t*-test for normally distributed values, or the Mann–Whitney test for non-normally distributed values. One-way ANOVA was used when comparing multiple groups, followed by Tukey’s multiple comparisons for *post-hoc* test. Data were presented as mean ± standard deviation (SD) or mean ± interquartile range as appropriate. Statistical significance was defined as *p* < 0.05.

## Results

### The effect of low-dose long-term aspirin on laser-induced choroidal neovascularization

**FIGURE 1:**
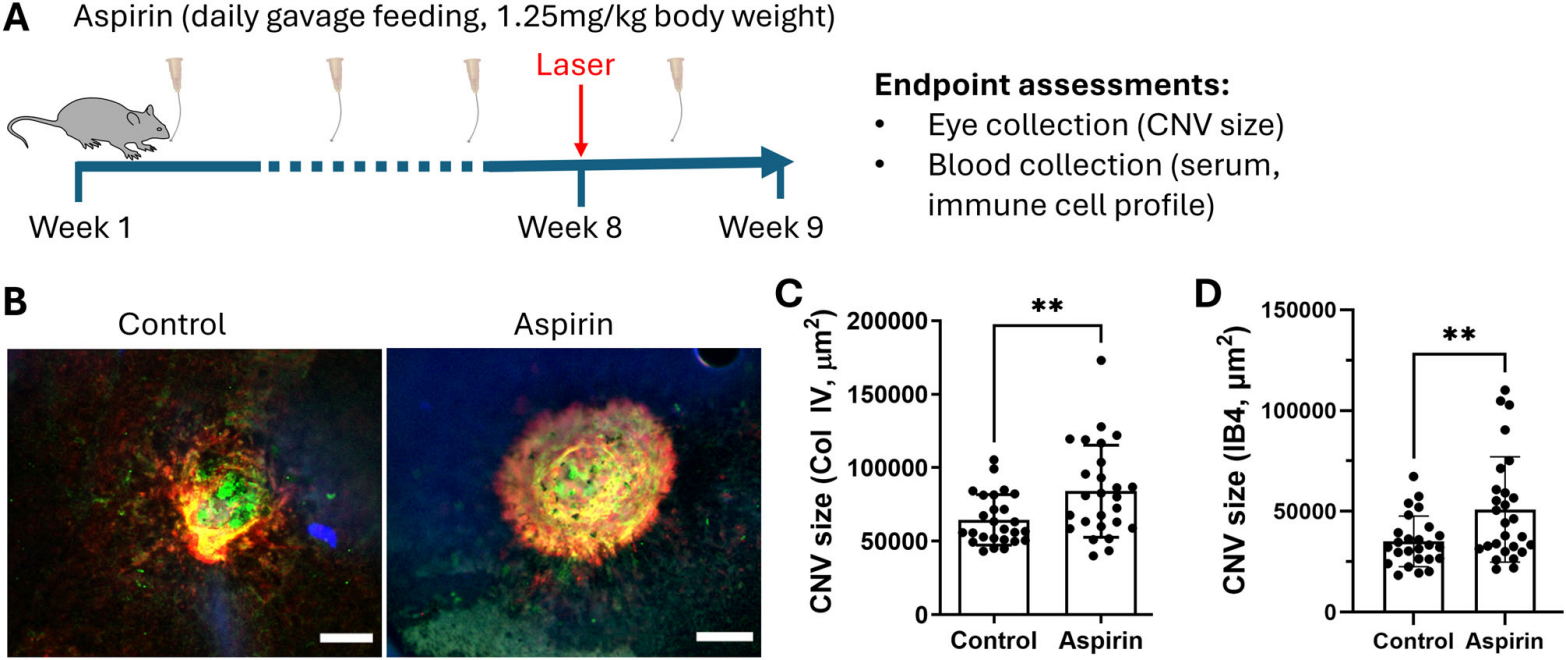
The effect of low-dose long-term aspirin on laser-induced choroidal neovascularization. **(A)** Schematic diagram showing study design. **(B)** Representative images showing collagen IV (red) and iso-lectin B4 (IB4) (green) staining in CNV lesions from vehicle control and aspirin-treated mice, scale bar = 50 μm. **(C)** Dot/bar graph showing collagen IV^+^ CNV lesion in the vehicle and aspirin-treated groups. **(D)** Dot/bar graph showing IB4^+^ CNV lesion in the vehicle and aspirin-treated groups. Mean ± SD, *n* = 26 lesions (from 8 eyes), **P < 0.01, Welch’s *t*-test.

To understand the impact of low-dose and long-term aspirin intake on neovascular AMD, mice were treated with 1.25 mg/kg (gavage feeding) of aspirin for 8 weeks and then subjected to laser-induced CNV (Figure 1A). Our results show that the CNV size (identified by collagen IV (Col IV, red) and isolectin B4 (IB4, green) (Figure 1B) was significantly larger in aspirin-treated mice compared to vehicle-treated control mice (Figures 1B–D).

### The effect of aspirin on circulating immune cells

**FIGURE 2:**
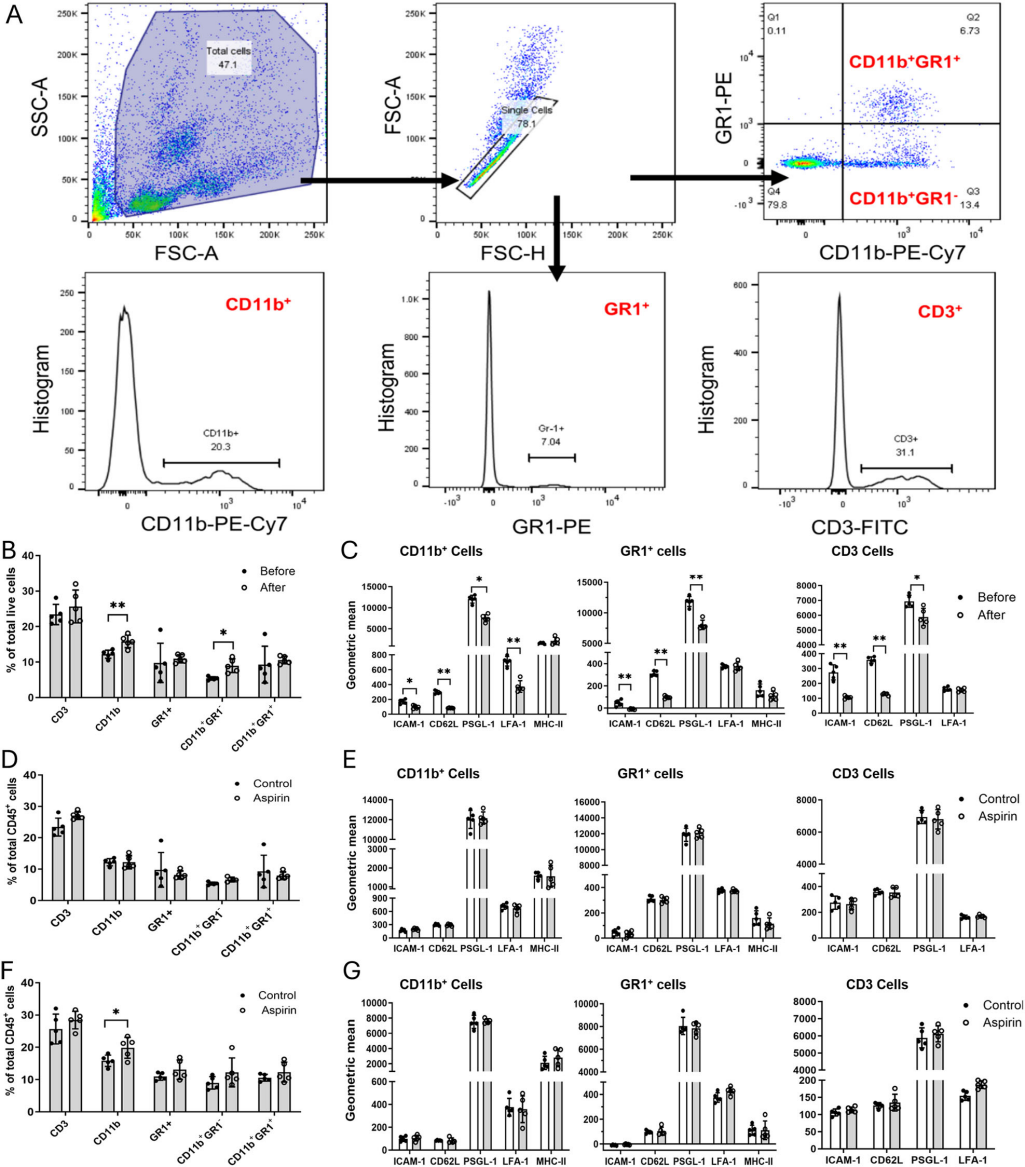
The effect of low-dose long-term aspirin on circulating immune cell constitution and activation in laser-induced choroidal neovascularization (CNV). **(A)** Gating strategies to identify different subsets of blood cells. **(B)** The percentage of different subsets of circulating immune cells before and after CNV in control mice. **(C)** The expression levels of cell surface molecules ICAM-1, CD62L, PSGD-1, LFA-1, and MHC-II in different subsets of circulating immune cells before and after laser-CNV in vehicle-treated control mice. **(D)** The percentage of different subsets of circulating immune cells in the non-lasered vehicle and aspirin-treated mice. **(E)** The expression levels of cell surface molecules ICAM-1, CD62L, PSGL-1, LFA-1, and MHC-II in different subsets of circulating immune cells in non-lasered control mice with or without aspirin treatment. **(F)** The percentage of different subsets of circulating immune cells in laser-induced CNV mice with/without aspirin treatment. **(G)** The expression levels of cell surface molecules ICAM-1, CD62L, PSGL-1, LFA-1, and MHC-II in different subsets of circulating immune cells in CNV mice with/without aspirin treatment. Mean ± SD, n = 5, *P < 0.05, **P < 0.01, Student *t*-test.

Inflammation is known to play an important role in CNV. To understand whether low-dose and long-term aspirin treatment promotes CNV through modulating systemic immune response to retinal laser injury, we evaluated circulating immune cell profiles using flow cytometry (Figure 2A).

First, we examined the impact of laser-induced CNV on circulating immune cells. After CNV induction, the populations of CD11b^+^ myeloid cells and CD11b^+^GR1^–^ monocytes were significantly increased (Figure 2B), and the expression levels of CD62L, ICAM-1, and PSGL-1 were significantly reduced in CD11b^+^ myeloid cells, GR1^+^ neutrophils, and CD3 T cells (Figure 2C). The expression of LFA-1 in CD11b^+^ myeloid cells was also reduced after CNV induction. The expression levels of MHC-II did not change before and after CNV induction (Figure 2C). Our results suggest that retinal laser injury increases the circulating innate immune cell population and reduces cell surface adhesion molecule expression.

Eight weeks of aspirin treatment in non-lasered mice affected neither the population of circulating immune cells (Figure 2D) nor the expression of cell surface adhesion molecules (Figure 2E). The treatment, however, significantly increased the population of CD11b^+^ cells in CNV mice (Figure 2F). CNV-mediated down-regulation of adhesion molecules was not affected by aspirin treatment (Figure 2G).

Taken together, our results suggest that long-term low-dose aspirin treatment enhanced laser-induced CNV-mediated increment in CD11b^+^ myeloid cells.

### The effect of aspirin on bone marrow-derived macrophages

**FIGURE 3:**
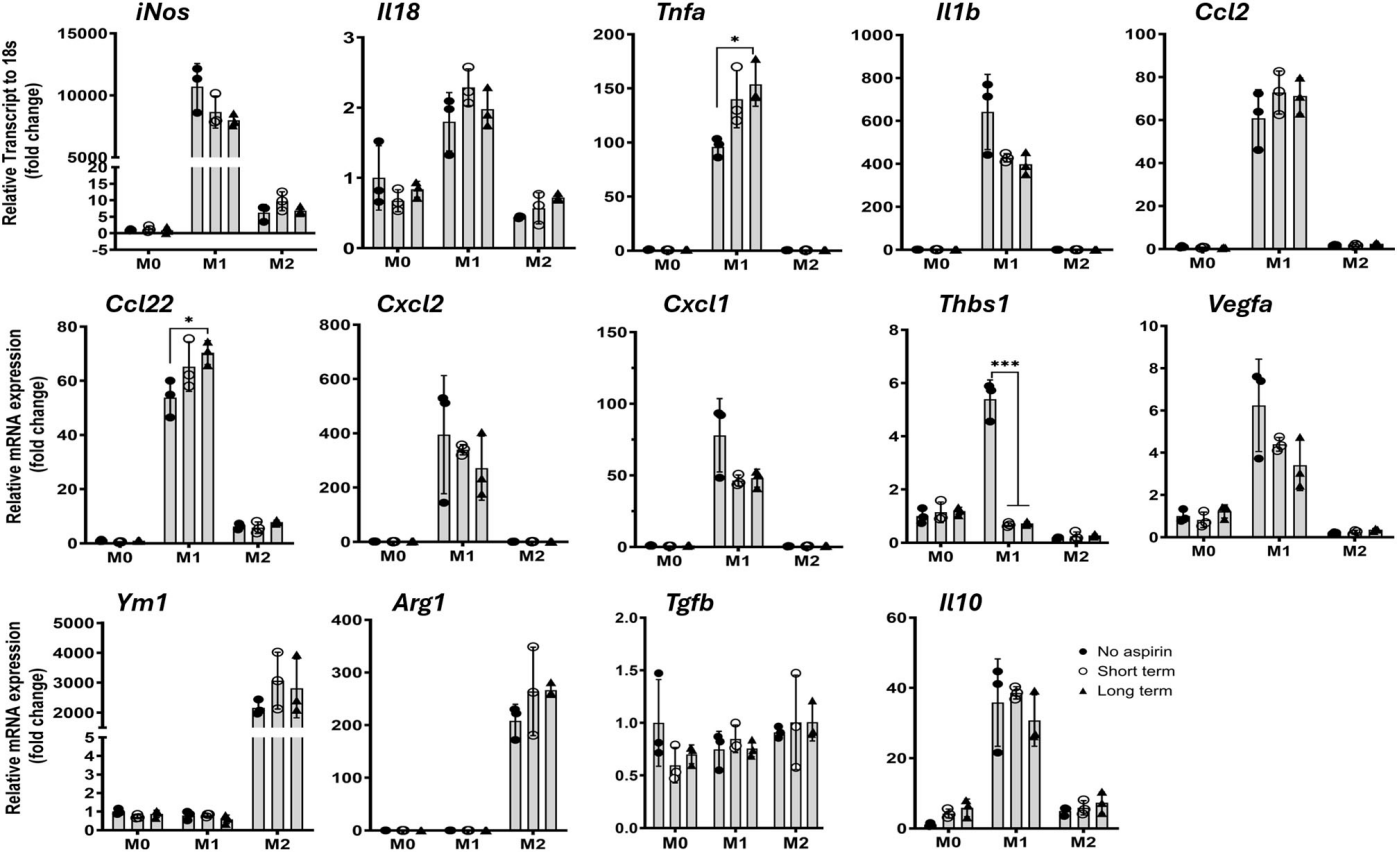
The effect of low-dose aspirin on cytokine gene expression in bone marrow-derived macrophage (BMDM). Mouse BMDMs were pretreated with aspirin (10 μM) for 1 h (short term) or 48 h (long term) before polarizing into M1 (LPS + IFNγ) or M2 (IL-4) for 16 h. The cells were then processed for qRT-PCR. Mean ± SD, *n* = 3, *P < 0.05, ***P < 0.001 by One-way ANOVA with Tukey’s multiple comparison.

To further understand how aspirin affects myeloid cells, we treated bone marrow-derived macrophages (BMDMs) with 10 μM of aspirin for 1 h (short-term) or 48 h (long-term) before polarizing into classically activated M1 (with LPS + IFNγ) or alternatively activated M2 (with IL-4) cells. Real-time PCR showed that M1 macrophages expressed high levels *of iNos, Il18, Tnfa, Il1b, Ccl2, Ccl22, Cxcl2, Cxcl11*, and *Thbs1*, whereas M2 macrophages expressed high levels of *Ym1* and *Arg1* (Figure 3). Interestingly, the expression of *Vegfa* and *Il10* was also increased in M1 macrophages, and the expression of *Tgfb* was not altered in M1 and M2 macrophages compared to naïve M0 cells (Figure 3). Aspirin treatment (1 and 48 h) did not affect the expression of any cytokine genes in M0 and M2 macrophages. Long-term aspirin treatment significantly increased the expression of *Tnfa* and *Ccl22* but reduced the expression of *Thbs1* in M1 macrophages (Figure 3).

**FIGURE 4:**
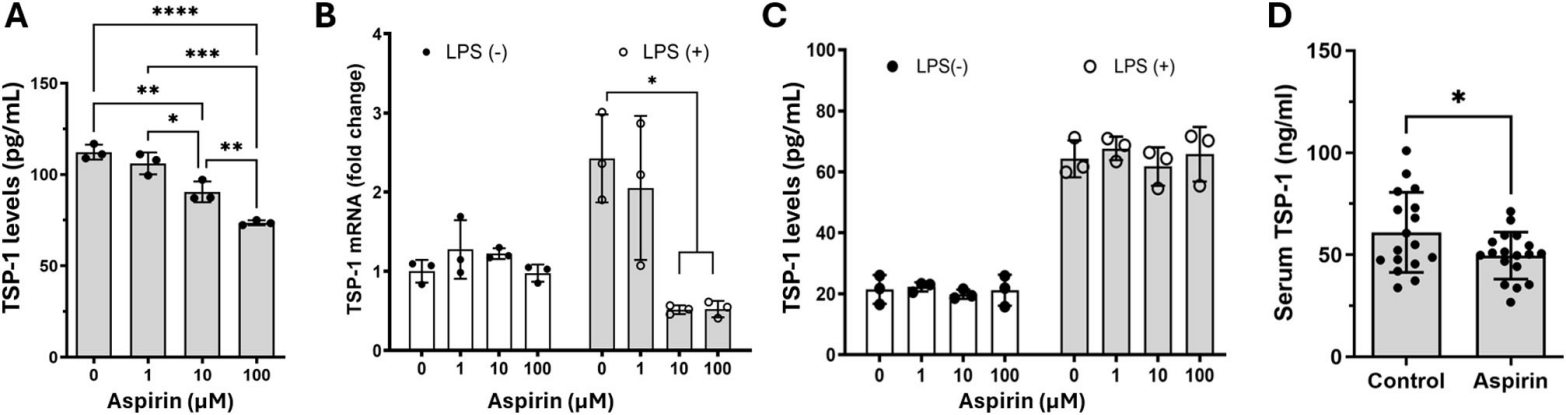
The effect of low-dose aspirin on the expression and secretion of TSP-1 in bone marrow-derived macrophage (BMDM), retinal pigment epithelial (RPE), cells and mouse serum. **(A)** Mouse BMDMs were pretreated with different concentrations of aspirin 48 h before polarizing into M1 (LPS + IFNγ) for 16 h. The supernatants were collected for the measurement of TSP-1 by ELISA. **(B,C)** Mouse primary RPE cells were pretreated with different concentrations of aspirin for 48 h followed by LPS (50 ng/mL) incubation for 16 h. The cells were processed for qRT-PCR (B), and supernatants were collected for ELISA **(C)**. Mean ± SD, *n* = 3, *P < 0.05, **P < 0.01, ***P < 0.001, ****P < 0.0001 by One-way ANOVA with Tukey’s multiple comparison. **(D)** TSP-1 levels in the serum from control and aspirin-treated mice (8 weeks of treatment). *N* = 17∼18, *P < 0.05, unpaired *t*-test.

The significant reduction of *Thbs1* gene expression by aspirin caught our attention as its protein, thrombospondin-1 (TSP-1), is known to have diverse roles in wound healing, angiogenesis, and inflammation (Kaur and Roberts, 2024; Liu et al., 2024). We further investigated the effect of aspirin on TSP-1 production in BMDMs and primary mouse RPE cells. Aspirin treatment dose-dependently reduced TSP-1 production in BMDMs (Figure 4A). Under normal culture conditions, mouse RPE cells expressed (Figure 4B) and produced (Figure 4C) low levels of TSP-1 and the expression was upregulated by LPS treatment (50 ng/mL, Figures 4B,C). Aspirin treatment did not affect TSP-1 production in RPE cells, although the mRNA expression was reduced by the high doses of aspirin (10 and 100 μM) in LPS-treated RPE cells (Figure 4B). To further confirm the suppressive effect of aspirin on TSP-1 production, we measured the TSP-1 levels in the serum of mice treated with 1.25 mg/kg for 8 weeks (Figure 1). We found that mice with aspirin treatment had significantly lower levels of TSP-1 compared to vehicle-treated mice (Figure 4D).

Our results suggest that low-dose, long-term aspirin treatment reduces TSP-1 production in disease conditions by downregulating circulating immune cells, in particular, myeloid cell *Thbs1* expression. Myeloid cell-derived infiltrating macrophages critically contribute to laser-induced CNV (Espinosa-Heidmann et al., 2003; Sakurai et al., 2003). Co-staining of RPE/choroidal wholemounts with Iba-1 and TSP-1 showed that 52.6% of Iba-1^+^ phagocytes co-expressed TSP-1, accounting for 63.2% of TSP-1^+^ cells in the CNV lesion (Supplementary Figures 1A,B), indicating that Iba-1^+^ phagocytes may be a major source of TSP-1 in CNV.

### The effect of low-dose, long-term aspirin on laser-induced CNV in Thbs-1 knockout mice

**FIGURE 5:**
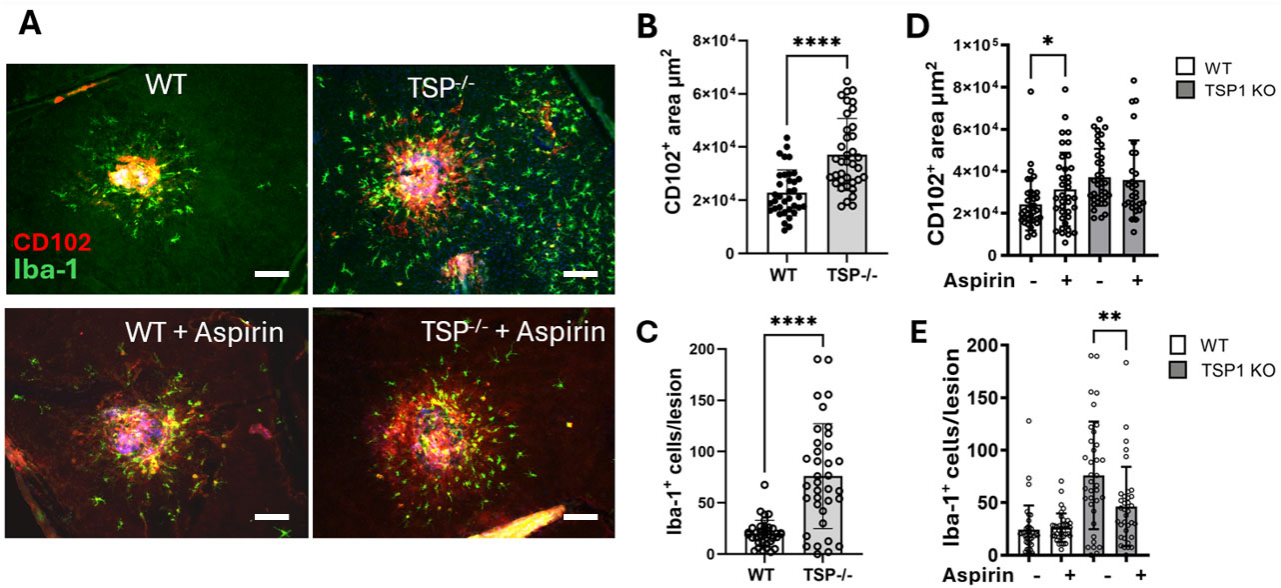
The effect of low-dose long-term aspirin on laser-induced choroidal neovascularization (CNV) in WT and TSP-1 KO mice. WT and TSP-1 KO mice were treated with aspirin (18 μg/mL) in drinking water for 8 weeks and then subjected to laser-induced CNV. Eyes were collected 7 days later and processed for RPE/choroidal flatmount staining of CD102 (red) and Iba-1 (green). **(A)** Representative images showing CNV lesions and Iba-1^+^ in different groups, scale bar = 50 μm. **(B)** Dot/bar figure showing CNV lesions in WT and TSP-1 KO mice. **(C)** Dot/bar figure showing subretinal Iba-1^+^ cells in WT and TSP-1 KO mice with laser-induced CNV. **(D)** Dot/bar figure showing the effect of aspirin treatment on CNV lesions in WT and TSP-1 KO mice. **(E)** Dot/bar figure showing the effect of aspirin treatment on Iba-1^+^ cells in WT and TSP-1 KO mice. Mean ± SD, *n* = 36∼40 from 8∼10 eyes, *P < 0.05, **P < 0.01, ****P < 0.0001 by Mann-Whitney test, one-tailed **(D)** and two-tailed **(B,C,E)**.

To understand whether aspirin-mediated downregulation of TSP-1 contributes to severe CNV (Figure 1), we conducted laser-induced CNV in aspirin-treated Thbs-1 KO mice. We found that the Thbs-1 KO mice developed more severe CNV, accompanied by a significantly higher number of Iba-1^+^ phagocyte infiltration to and around the lesion than WT mice (Figures 5A–C).

Aspirin treatment increased laser-induced CNV in WT mice but not Thbs-1 KO mice (Figures 5A,D). Interestingly, the treatment significantly reduced the number of infiltrating Iba-1^+^ phagocytes in Thbs-1 KO mice but not WT mice (Figures 5A,E).

### The levels of TSP-1 and VEGF in nAMD patients with/without aspirin use

There were no significant differences in gender, history of AMD, history of cardiovascular disease or taking cardiovascular medication, history of hypertension, history of diabetes, body mass index (BMI), smoking habits between controls and nAMD patients. There were more nAMD patients taking vitamins or low-dose aspirin compared to controls (Table 3). A significantly higher number of nAMD patients with low-dose aspirin had a history of cardiovascular disease and were taking cardiovascular medication, and a history of diabetes (Supplementary Table 1).

**TABLE 3:** Demographic and clinical characteristics of nAMD patients and controls.

Characteristics	All (*n* = 213)	Controls (*n* = 43)	nAMD (*n* = 170)	*P*-value nAMD vs. control
Age [median (range)], years	79.4 (53–93)	74.0 (58–92)	80.1 (53–93)	**0.012** ^1^
Female sex [number (%)]	108 (51)	19 (44)	89 (52)	0.339^2^
Have history of AMD [number (%)]	45 (21)	6 (14)	39 (23)	0.213^2^
Have history of cardiovascular disease [number (%)]	55 (26)	9 (21)	46 (27)	0.456^2^
Have history of hypertension [number (%)]	130 (61)	23 (53)	107 (63)	0.330^2^
Have history of diabetes [number (%)]	27 (13)	2 (5)	25 (15)	0.083^2^
Body mass index (mean ± SD)	26.1 (4.5)	26.1 (5.1)	26.0 (4.2)	0.952^3^
Smoking status	89 (42)	20 (47)	69 (41)	0.601^2^
Non-smoker [number (%)]				
Former smoker [number (%)]	107 (50)	20 (47)	87 (51)	
Current smoker [number (%)]	16 (8)	2 (5)	14 (8)	
Taking cardiovascular medication [number (%)]	153 (72)	28 (65)	125 (74)	0.374^2^
Taking vitamins [number (%)]	48 (23)	3 (7)	45 (27)	**0.007** ^2^
Taking low-dose aspirin [number (%)]	69 (32)	5 (12)	64 (38)	**0.001** ^2^

^1^Mann-Whitney U test;

^2^Pearson’s chi-square test;

^3^Independent samples *t*-test; SD, Standard deviation; Bold indicate *P* < 0.05.

**FIGURE 6:**
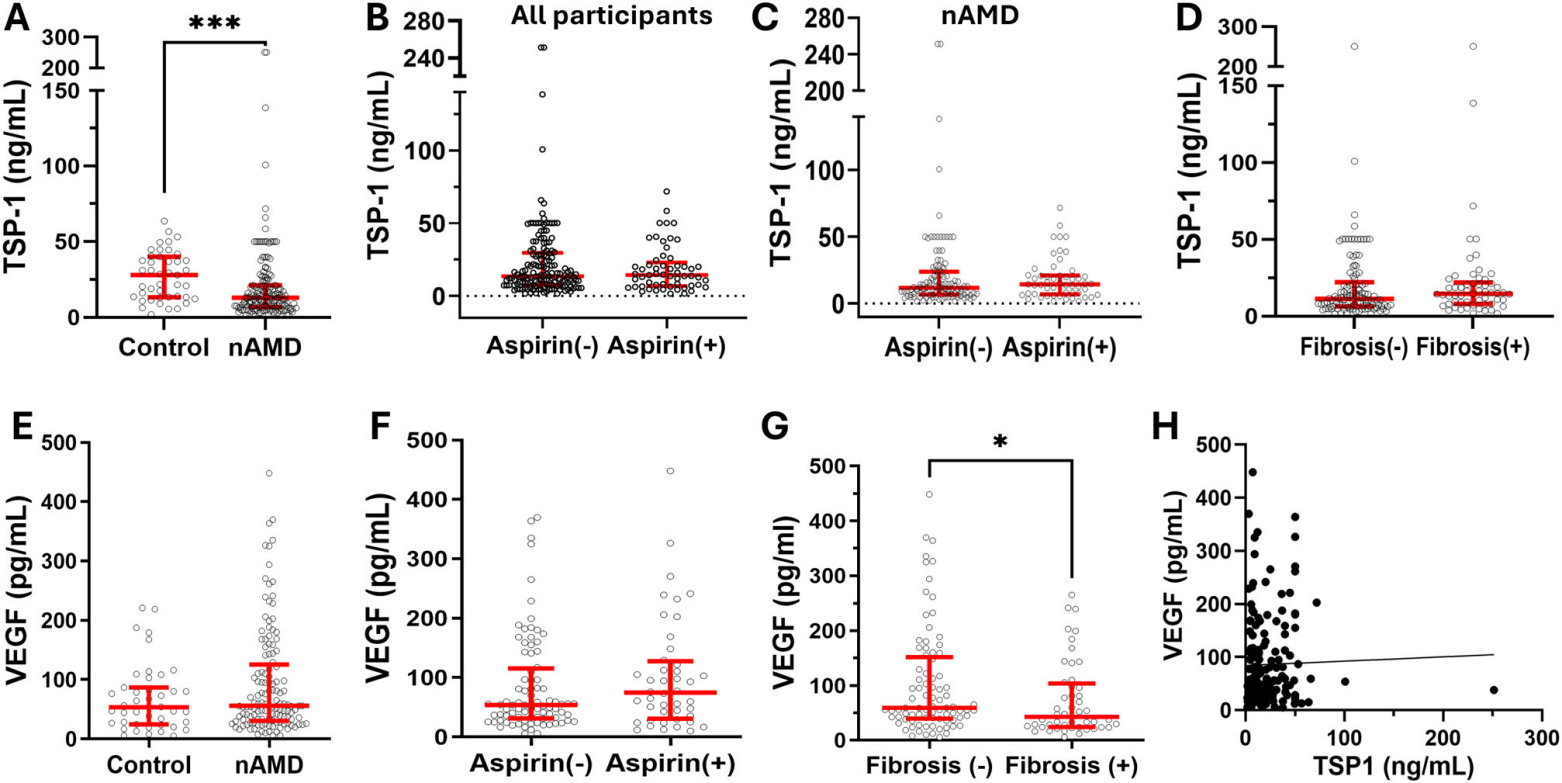
The serum levels of TSP-1 and VEGF in nAMD patients with and without aspirin use. Serum was collected from nAMD patients and healthy controls, and the levels of TSP-1 and VEGF were measured by ELISA. **(A)** The levels of TSP-1 in nAMD (*n* = 168) and controls (*n* = 43). **(B)** The levels of TSP-1 in all participants (regardless of AMD) with (*n* = 57) and without (*n* = 154) aspirin use. **(C)** The levels of TSP-1 in nAMD patients with (*n* = 53) and without (*n* = 115) aspirin use. **(D)** The levels of TSP-1 in nAMD with (*n* = 58) and without (*n* = 110) macular fibrosis. **(E)** The serum levels of VEGF in nAMD patients (*n* = 133) and controls (*n* = 43). **(F)** The levels of VEGF in nAMD with (*n* = 44) and without (*n* = 84) aspirin use. **(G)** The levels of VEGF in nAMD with (*n* = 48) and without (*n* = 85) macular fibrosis. **(H)** The correlation between serum levels of TSP-1 and VEGF in all participants (*n* = 174). Mean ± interquartile range, *P < 0.05, ***P < 0.001 by Mann-Whitney test.

To understand the clinical relevance of our results, we measured the TSP-1 levels in the serum of nAMD patients. We found that the serum level of TSP-1 was significantly lower in nAMD patients than in age-matched healthy controls (Figure 6A). This is in line with previous findings that wet AMD retina exhibited reduced TSP-1 immunolabeling (Uno et al., 2006; Obasanmi et al., 2023). When all participants (regardless of AMD) were analyzed together, the serum level of TSP-1 in aspirin users had a non-significant trend toward lower values compared with that in non-aspirin users (*p* = 0.077, *t* = 1.433, df = 194.9, unpaired *t*-test with Welch’s correction, Figure 6B). However, we did not detect any significant difference between patients who use and those who do not use aspirin (Figure 6C). TSP-1 is known to be involved in organ fibrosis (Xu et al., 2020). The serum level of TSP-1 in patients with and without macular fibrosis was comparable (Figure 6D). We previously reported that the serum level of VEGF was slightly higher in nAMD patients than in controls, although not statistically significant (Lechner et al., 2017) (Figure 6E). We further analyzed the serum VEGF levels in nAMD patients with and without aspirin but found no differences (Figure 6F). Interestingly, the serum level of VEGF was significantly lower in nAMD patients with macular fibrosis than in those without (Figure 6G). Neither the serum levels of VEGF and TSP-1 (Figure 6H) nor their levels and the number of intravitreal anti-VEGF injections were correlated (TSP-1: *R*^2^ = 0.02, *p* = 0.06, *n* = 168; VEGF: *R*^2^ = 0.005, *p* = 0.40, *n* = 133).

## Discussion

In this study, we show that low-dose long-term aspirin use enhanced the severity of laser-induced CNV. Mechanistically, aspirin treatment increased the population of circulating CD11b^+^ cells and reduced TSP-1 production in myeloid cells. The serum level of TSP-1 was significantly lower in aspirin-treated mice compared to vehicle-treated controls. TSP-1 is a matricellular glycoprotein involved in angiogenesis, inflammation, and cancer (Ren et al., 2006; Lawler, 2002). Previous studies have demonstrated that TSP-1 plays a critical role in regulating retinal/subretinal inflammation and angiogenesis (Beguier et al., 2020; Housset and Sennlaub, 2015; Chen et al., 2012; Zamiri et al., 2005). TSP-1 expression was impaired in the macular tissue of AMD patients, particularly in the CNV lesion (Uno et al., 2006). We found that nAMD patients had significantly lower serum levels of TSP-1 compared to age-matched healthy controls (Figure 6), suggesting that insufficient TSP-1 production may be a risk factor for nAMD. Our results highlight the potential risk of low-dose and long-term aspirin use in worsening the severity or progression of nAMD.

Although nAMD patients had significantly lower levels of TSP-1 compared to controls (Figure 6), unlike our observation in aspirin-treated mice, the serum levels of TSP-1 in nAMD patients who used aspirin were comparable to those in patients who did not (Figure 6). Several reasons may contribute to the discrepancy. First, the information on aspirin use was based on data collected from questionnaire sheets, which did not provide information on the duration of or patient compliance with aspirin use. Second, the potential confounders of TSP-1, such as genetics, cardiovascular diseases, chronic inflammatory diseases, and the use of other nonsteroidal anti-inflammatory drugs (e.g., ibuprofen), etc., were not controlled. In our study, the percentage of people with diabetes or cardiovascular diseases was higher in patients with aspirin. When all participants were analyzed together (regardless of AMD), the serum level of TSP-1 in aspirin users was marginally lower than that in non-aspirin users (Figure 6B). Third, although we endeavored to recruit age-matched controls, a significant age difference persisted between nAMD patients and controls (Table 3). Further large-scale randomized controlled studies will be needed to understand whether low-dose and long-term aspirin use reduces TSP-1 production in patients.

In this study, aspirin treatment affected neither the circulating immune cell profile/cell surface adhesion molecule expression in non-lasered control mice, nor the cytokine gene expression in naïve macrophages and RPE cells *in vitro*. The suppressive effect of aspirin on TSP-1 expression and production was only observed in laser-induced CNV mice and LPS-treated macrophages. Our results suggest that low-dose long-term aspirin use alters immune cell response in inflammatory and angiogenic conditions. The expression of the TSP-1 gene (*THBS*) is upregulated in immune cells, particularly myeloid-derived cells, such as macrophages, dendritic cells, and neutrophils, under inflammatory or ischemic conditions (Lopez-Dee et al., 2011). This is in line with our observation in the laser-induced CNV, whereby a high level of TSP-1 was detected in Iba1^+^ cells (Supplementary Figure 1). As a cyclooxygenase (COX) inhibitor, aspirin can reduce prostaglandin production, and prostaglandins are normally produced under inflammatory conditions. Previous studies have shown that prostaglandin E2 (PGE2) (Spicer et al., 2022) and F2α (PGF2) (Farberov et al., 2019) can upregulate *THBS* gene expression and induce TSP-1 production in inflammatory macrophages. On the other hand, TSP-1 can suppress prostaglandin production as a negative feedback loop. Macrophages produce PGE2 when they are activated (Ulmann et al., 2010). This may explain our observation that aspirin only affected cytokine expression (including TSP-1 production) in LPS-treated (not naïve) macrophages and laser-induced CNV (not normal) mice.

The lack of significant effect of aspirin on immune cells under normal conditions may also explain the clinical observation that low-dose and long-term aspirin use did not affect the incidence of all AMD but increased the risk of late-stage and nAMD (Ye et al., 2014; Liew et al., 2013; de Jong et al., 2012; Klein et al., 2012; Yan et al., 2022). A recent study showed that circulating innate immune cell activation positively correlated with the severity of AMD (Ojaghi et al., 2024). We and others have reported various types of circulating immune cell activation in advanced AMD (e.g., nAMD) (Chen et al., 2016; Lechner et al., 2017, 2015; Subhi et al., 2019b; Subhi et al., 2019a; Krogh Nielsen et al., 2017). Patients with nAMD constantly experience vascular leakage (macular edema) and/or hemorrhage, which would induce more severe inflammation compared to early and intermediate AMD. Since aspirin preferentially affects active immune cells, it may have limited effects on the onset of AMD but can profoundly affect late-stage and/or active nAMD.

It should be noted that, in addition to its role in immune cell alteration, aspirin can also increase the risk of hemorrhage in different organs, such as the gastrointestinal system (Mahady et al., 2021; García Rodríguez et al., 2019). A previous study reported a significant increase in intracranial bleeding with daily low-dose aspirin use, but no substantial reduction in ischemic stroke (Cloud et al., 2023). Macular hemorrhage and edema are common features of nAMD. The beneficial (reducing cardiovascular risk) versus detrimental (promoting nAMD progression and severity) of low-dose and long-term aspirin use in nAMD should be thoroughly evaluated in clinical settings.

It should be noted that low-dose aspirin is often prescribed to the elderly; the use of young adult mice in the study is a limitation. Aging leads to chronic low-grade systemic immune activation (“inflammaging”) and induces retinal para-inflammation (Chen et al., 2019; Chen and Xu, 2015). This altered immune system might be more sensitive to aspirin-mediated down-regulation of TSP-1, particularly in AMD patients whose TSP-1 expression is already impaired in the retina (Uno et al., 2006; Obasanmi et al., 2023).

## Conclusion

This study shows that low-dose and long-term aspirin use increases the severity of laser-induced CNV by downregulating TSP-1 in myeloid cells. The study highlights the need to carefully evaluate the potentially harmful effects of low-dose and long-term aspirin use on ocular diseases such as nAMD in large-scale epidemiological studies.

## Data Availability

The original contributions presented in this study are included in this article/Supplementary material, further inquiries can be directed to the corresponding authors.
