# Supplementary material for: Long-term low-dose aspirin promotes laser-induced choroidal neovascularization through suppressing TSP-1 expression
Source: Front Cell Neurosci. 2025 Dec 5;19:1716229. doi: 10.3389/fncel.2025.1716229 (PMC12714992; doi:10.3389/fncel.2025.1716229)
Supplement: Supplementary file 1 [file Table_1.docx]

**Supplementary Figure S1**

**
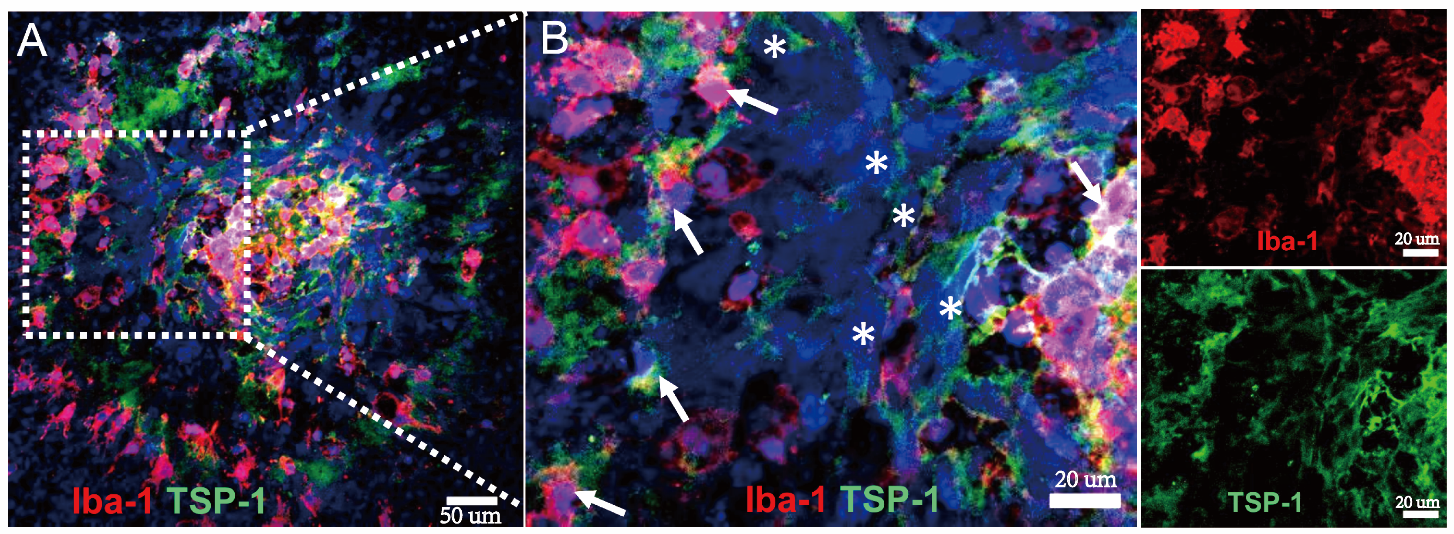
**

**Figure S1.** TSP-1 expression in mouse eyes with choroidal neovascularisation (CNV). (A) Representative confocal image showing Iba-1 (red) and TSP-1 (green) expression inside and around the CNV lesion. scale bar = 50 µm. (B) Magnified area of box in (A) showing Iba-1^+^TSP-1^+^ cells (arrows), Iba-1^-^TSP-1^+^ cells (asterisks) in CNV lesion, scale bar = 20 µm.

**Supplementary Table S1**

**Table S1.** Demographic and clinical characteristics of nAMD patients with/without low-dose aspirin intake.

|  | **nAMD**  **(n = 170)** | **Aspirin (-)**  **(n = 106)** | **Aspirin (+)**  **(n = 64)** | ***P* value**  **Aspirin (-) vs (+)** |
| --- | --- | --- | --- | --- |
| Age (median (range)), years | 80.1 (53-93) | 80.6 (56-93) | 79.9 (53-93) | 0.067^1^ |
| Female sex (number (%)) | 89 (52) | 59 (56) | 30 (47) | 0.266^2^ |
| Have history of AMD (number (%)) | 39 (23) | 24 (23) | 15 (23) | 0.931^2^ |
| Have history of cardiovascular disease (number (%)) | 46 (27) | 16 (15) | 30 (47) | **<0.001^2^** |
| Have history of hypertension (number (%)) | 107 (63) | 61 (58) | 46 (72) | 0.061^2^ |
| Have history of diabetes (number (%)) | 25 (15) | 11 (10) | 14 (22) | **<0.040^2^** |
| Body Mass Index (mean ± SD) | 26.0 (4.2) | 26.3 (4.2) | 25.4 (4.4) | 0.318^3^ |
| Smoking status |  |  |  | 0.987^2^ |
| Non-smoker (number (%)) | 69 (41) | 43 (41) | 26 (41) |  |
| Former smoker (number (%)) | 87 (51) | 54 (51) | 33 (51) |  |
| Current smoker (number (%)) | 14 (8) | 9 (8) | 5 (8) |  |
| Taking cardiovascular medication (number (%)) | 125 (74) | 70 (66) | 55 (86) | **0.004^2^** |
| Taking vitamins (number (%)) | 45 (27) | 27 (25) | 18 (28) | 0.704^2^ |
| Fibrosis present (number (%)) | 58 (38) | 37 (35) | 21 (33) | 0.780^2^ |
|  | | | | |

^1^ Mann Whitney U test; ^2^ Pearson’s chi-square test;^3^ Independent samples t-test; SD: Standard deviation; **Bold** *P*<0.05
